# Primary culture and endocrine functional analysis of Leydig cells in ducks (*Anas platyrhynchos*)

**DOI:** 10.3389/fendo.2023.1195618

**Published:** 2023-06-06

**Authors:** Xiaoya Chu, Aiman Javed, Muhammad Faizan Ashraf, Xiuge Gao, Shanxiang Jiang

**Affiliations:** ^1^ Engineering Center of Innovative Veterinary Drugs, Center for Veterinary Drug Research and Evaluation, Ministry of Education (MOE) Joint International Research Laboratory of Animal Health and Food Safety, College of Veterinary Medicine, Nanjing Agricultural University, Nanjing, Jiangsu, China; ^2^ Department of Psychiatry & Behavioral Sciences, King Edward Medical University, Lahore, Punjab, Pakistan; ^3^ Department of Basic Sciences, Fatima Memorial Hospital (FMH) College of Medicine & Dentistry, Lahore, Pakistan

**Keywords:** primary culture, testosterone, Leydig cell, duck (*Anas platyrhynchos*), isolation

## Abstract

Testicular Leydig cells (LCs) are the primary known source of testosterone, which is necessary for maintaining spermatogenesis and male fertility. However, the isolation, identification, and functional analysis of testosterone in duck LCs are still ambiguous. The aim of the present study was to establish a feasible method for isolating highly purified primary duck LCs. The highly purified primary duck LCs were isolated from the fresh testes of 2-month-old ducks via the digestion of collagenase IV and Percoll density gradient centrifugation; hematoxylin and eosin (H&E), immunohistochemistry (IHC) staining, ELISA, and radioimmunoassay were performed. Results revealed that the LCs were prominently noticeable in the testicular interstitium of 2-month-old ducks as compared to 6-month-old and 1-year-old ducks. Furthermore, IHC demonstrated that the cultured LCs occupied 90% area of the petri dish and highly expressed 3β-HSD 24 h after culture (hac) as compared to 48 and 72 hac. Additionally, ELISA and radioimmunoassay indicate that the testosterone level in cellular supernatant was highly expressed in 24 and 48 hac, whereas the testosterone level gradually decreased in 72 and 96 hac, indicating the primary duck LCs secrete testosterone at an early stage. Based on the above results, the present study has effectively developed a technique for isolating highly purified primary duck LCs and identified its biological function in synthesizing testosterone.

## Introduction

Leydig cell (LC) is a kind of interstitial cell present in the loose connective tissues between seminiferous tubules in the testis (1). The main function of the LC is synthesizing and secreting androgen, and more than 90% of testosterone in the body originates from LCs (2). The testosterone synthesized by LC is proved to be regulated by the hypothalamic–pituitary–gonadal (HPG) axis (3), during which the secretion of gonadotrophin-releasing hormone (GnRH) acted on the anterior pituitary and then promoted the synthesis and secretion of luteinizing hormone (LH) and follicle-stimulating hormone (FSH). Additionally, age-related decline in the function of the hypothalamic–pituitary gland is one of the key factors contributing to the decline in serum testosterone levels (2, 4).

LC produces testosterone, which serves a variety of physiological functions in the body at various developmental phases (5). It could not only promote the development of gonads (6) but also sustain spermatogenesis and sperm maturation within the male reproductive system (7). Without testosterone, spermatogenesis would suspend at meiosis II, which leads to the decline of spermatogenic cells after meiosis and the lack of elongated spermatids (8). Testosterone supplementation could be used to restore normal testosterone levels, which stimulate the process of spermatogenesis (9). In previous studies, testosterone has been proven to possess a variety of physiological functions. First, testosterone could induce sex differentiation and development of the male reproductive system during the embryonic phase. However, due to testosterone, the undifferentiated gonads will convert into male testes (10). The masculinization of the genital ducts as well as external genitalia has been induced by testosterone, and it promotes testicular descent to the scrotum (11). In male infants, the secretion of testosterone is at a very low level and remains constant up to the onset of puberty. At the early stage after birth, the levels of luteinizing and follicle-stimulating hormone are comparatively low, which causes minimal testosterone production levels (12). Testosterone could induce secondary sexual characteristics at puberty. LCs secrete testosterone again upon the start of puberty, and the testes develop and begin to produce sperm in response to testosterone stimulation (13). Meanwhile, the accessory sexual glands also develop secretory activity; furthermore, testosterone could maintain spermatogenesis after sexual maturity. Serum testosterone levels sharply decline and spermatogenesis is inhibited after the removal of the testis in adulthood (14, 15). Testosterone could promote the metabolism and development of the body; stimulates the growth of bone, skeletal muscle, hair, and skin; and promotes the production of red blood cells (16). Consequently, testosterone could not only sustain spermatogenesis but also be widely involved in the metabolic activities of the body.

As indicated previously, multitudinous inherent and exterior factors have been correlated in reproductive diseases, which are induced by the disorder in testosterone synthesis and secretion. LC is not only the cell that synthesizes and secretes testosterone but also the target cell of testosterone (17). Testosterone regulates the development of LCs via the differentiation into adult Leydig cells (ALCs) during the development of male livestock and poultry (18). Innately, such malformation of reproductive organs or salpingemphraxis and acquired breeding disorder (for example, cryptorchidism and testicular dysgenesis syndrome) could be owing to the defect in testosterone synthesis and secretion (19). Alternatively, heat shock affects the endocrine system (especially male adult domestic animals) and significantly declines reproductive capacity through testosterone reduction (20). In addition, testosterone synthesis in chicken could also be regulated by different photoperiods (21). With regard to the virus–male reproductive system interaction, the infection of Zika virus (ZIKV), an emerging mosquito-borne flavivirus, could lead to testicular atrophy and orchitis, which are probably caused by LC infection and a concomitant decline in testosterone synthesis (22). In addition to ZIKV, Mumps virus (MuV) (23) and Japanese encephalitis virus (JEV) (24) are also closely correlated with suppressed testosterone synthesis in LCs and impaired male fertility. Additionally, the influence of environmental stimulus on human health, especially the reproductive system, has attracted more and more extensive attention. Among them, PM_2.5_ has been proven to induce orchitis via NF-κB signal pathway activation and is further discovered to have the reliving effect of aspirin in orchitis (25). Endocrine-disrupting compounds (EDCs) are another environmental stimulus that could impair the development of the male reproductive system by androgen disruption and inhibit steroidogenesis in LCs as well (26). Moreover, the effects of bisphenol A (BPA) in attenuating testosterone synthesis and secretion are thoroughly studied. Consequently, the guarantee for the ability of testosterone synthesis and secretion in LCs is of significant value (27). Furthermore, the illustration of mechanisms underlying testosterone synthesis and therapies *in vitro* call for the isolation and primary culture of LCs but has caught limited attention.

Currently, the studies related to the isolation and culture of LCs were mainly focused on mice (28), rats (29), pigs (30), sheep (31), cows (32), and other mammals (33). In poultry, collagenase II digestion combined with differential centrifugation was applied in the isolation of rooster LCs, and passage was used in its purification (34). In contrast, the isolation and identification of highly purified primary LCs in ducks have received very little attention. Accordingly, in the present study, we aimed to establish a feasible method for isolating highly purified primary duck LCs and analyze their ability for testosterone synthesis and secretion.

## Materials and methods

### Animals

Eighteen male ducks (*Anas platyrhynchos*) aged 2 months, 6 months, and 1 year (six in each group) were purchased from Nanjing Qizai Biological Co., Ltd. Moreover, ten 2-month-old ducks were used in the culture of LCs. After adaptive feeding for 48 h, the testes of both sides were immediately separated and sterilized with 75% ethanol. The procedures involving the care and use of animals in the experiment had been approved by the Animal Research Institute Committee guidelines of the Nanjing Agriculture University, China. The Science and Technology Agency of Jiangsu Province and Nanjing Agricultural University Veterinary College approved the sampling procedures with approval ID SYXK (SU) 2010-0005.

### Hematoxylin and eosin staining

Fresh testes of ducks were first fixed in a modified Davidson’s solution for 24 to 72 h and then transferred to 4% paraformaldehyde for 48 h. After that, six testes in every group were embedded in paraffin after trimming, dehydration, and substitution in xylene. Moreover, the cultured LCs were fixed with 4% paraformaldehyde for 30 min after the supernatant was discarded. The paraffin blocks were sectioned with a thickness of 5 μm and stained with hematoxylin and eosin. Finally, the slides were examined microscopically by a light microscope (DP73, Olympus, Tokyo, Japan).

### Immunohistochemistry staining

The paraffin sections and cultured LCs were incubated with rabbit polyclonal anti-HSD3B1 antibodies (ab55268, Abcam, Massachusetts, USA). After washing, the slides were incubated with biotinylated goat anti-rabbit IgG (ab64256, Abcam, Massachusetts, USA) for 1 h. After rinsing in phosphate-buffered saline (PBS), DAB (ab64261, Abcam, Massachusetts, USA) was used for the visualization of peroxidase activity. The slides were examined microscopically by a light microscope (DP73, Olympus, Tokyo, Japan).

### Isolation and primary culture of duck Leydig cells

The LCs were first sterilized at the surface of the testes with 75% ethanol. Then, the connective tissue and albuginea were stripped from the testes and chopped into pieces with sterilized scissors. The collagenase II (1148090, Sigma-Aldrich, Saint Louis, MO, USA) with a concentration of 1 mg/ml was added and underwent shock digestion at 37°C for 1–1.5 h. Digestion was terminated by DMEM/F12 medium when the seminiferous tubule was loosened. The mixture was filtered through a 100 mesh stainless steel filter, the liquid was collected and centrifuged at 409 g for 10 min, and then the liquid was removed. The step was repeated with a 200 mesh stainless steel filter, and the cell suspension into Percoll (60%, 34%, 26%) was centrifuged at 728 g for 30 min. The third layer of the cell zone, which was counted from the top to the bottom, was taken out by the syringe. The DMEM/F12 medium was added, mixed with the cells, and centrifuged at 409 g for 10 min. DMEM/F12 medium containing 10% fetal bovine serum (FBS) and 1% penicillin–streptomycin was added to the purified cells for cell suspension. Finally, cell suspension of approximately 5 × 10^5^ cells/ml was incubated in 5% CO_2_ at 37°C.

### Total RNA extraction

RNA of testis tissue (small species) and LCs (three samples in each group) were extracted using TRIzol Reagent (T9424, Sigma-Aldrich, Saint Louis, MO, USA). RNA concentration was measured using NanoDrop 1000 spectrophotometer (ND-1000, Thermo Fisher Scientific, Massachusetts, USA). The ratio of A260/A280 was applied to detect the RNA integrity, and the threshold value was set between 1.8 and 2.0. The extracted RNA was immediately applied for cDNA synthesis or stored at −80°C.

## ELISA analysis

The testosterone levels of LC supernatant were detected using Duck Testosterone ELISA Kit (Jiangsu Meimian Industrial Co., Ltd., China). The cellular supernatant testosterone levels were detected following the manufacturers’ instructions. The cellular supernatant was centrifuged at 1,000 *g* for 20 min and mixed with the reagent in 96-well plates. After incubation at 37°C for 30 min, it was washed with PBS two to three times. Then, the enzyme-labeled reagent was added and incubated at 37°C for 30 min. Optical density (OD) at 450 nm was applied using Microplate Reader (51119700DPC, Thermo Fisher Scientific, Massachusetts, USA).

### Radioimmunoassay

The testosterone levels of the LC supernatant were detected using an Iodine [^125^I] Testosterone Radioimmunoassay Kit (B10B, Beijing North Institute of Biotechnology Co., Ltd., China). The cellular supernatant testosterone levels were detected following the manufacturers’ instructions.

### Real-time quantitative polymerase chain reaction

The first-strand cDNA was synthesized using HiScript III RT SuperMix for qPCR (R323-01, Vazyme, Nanjing, China). SYBR Green PCR Master Mix (Q111-02, Vazyme, Nanjing, China) was applied to real-time fluorescence quantitative PCR assays via LightCycler 480 (Roche, Switzerland). The PCRs were first conducted under 94°C for 5 min and then followed 40 cycles’ amplification of denaturation at 95°C for 30 s, 60°C for 30 s for the annealing, and 60°C to 95°C for the melting curves. The primers for the following genes were synthesized using Primer3 Input (version 0.4.0) software, and details are attached in **Supplementary Table 1**. β-Actin was used as an internal control. The relative changes in gene expression between different groups were calculated using the 2^−ΔΔCT^ method.

### Statistical analysis

All data were presented as mean ± standard error of the mean (SEM). The statistical data were obtained by importing data into SPSS for Windows version 22.0 statistical package (SPSS Inc., Chicago, IL, USA). The normality and the equality of variances of data were assessed by ANOVA. The data were considered statistically significant when p < 0.05.

## Results

### Morphological evaluations of LCs in the testicular interstitial tissues

**Figure 1:**
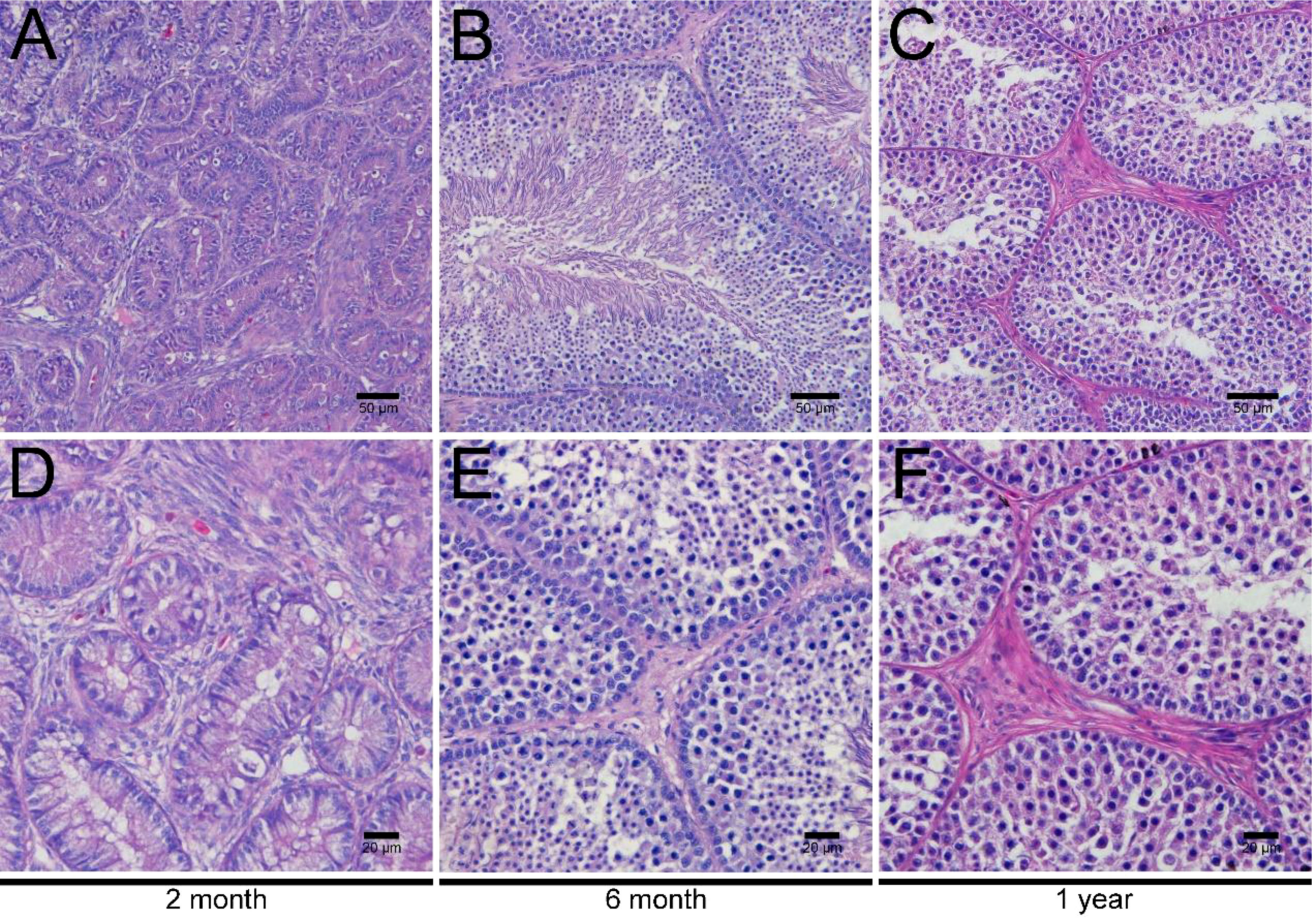
H&E staining of testes in ducks aged 2 months, 6 months, and 1 year. Low magnification of testes from ducks aged 2 months **(A)**, 6 months **(B)**, and 1 year **(C)**. High magnification of testes from ducks aged 2 months **(D)**, 6 months- **(E)**, and 1 year **(F)**.

**Figure 2:**
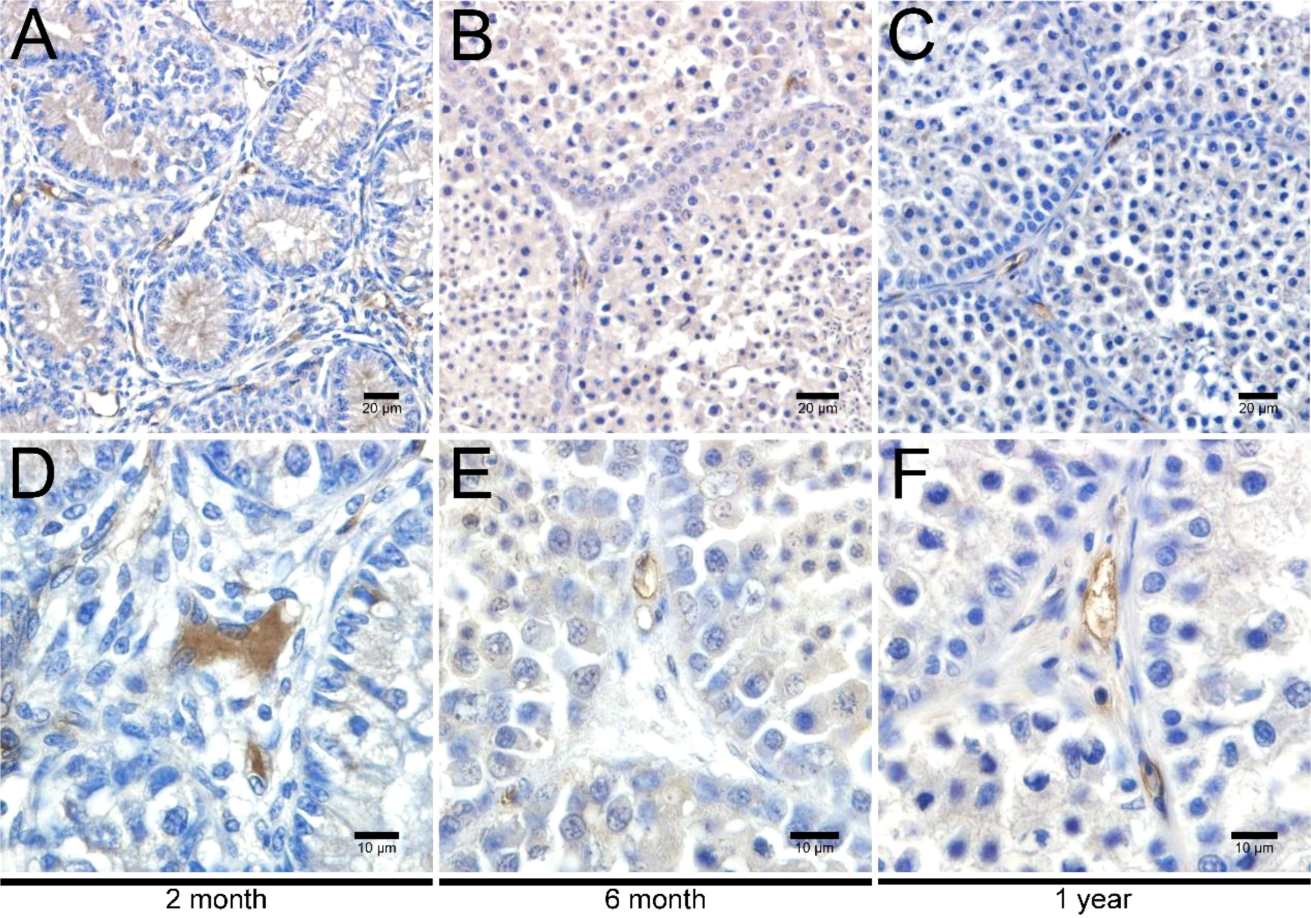
Immunohistochemical analysis of 3β-HSD in testes. Low magnification of testes from ducks aged 2 months **(A)**, 6 months **(B)**, and 1 year **(C)**. High magnification of testes from ducks aged 2 months **(D)**, 6 months **(E)**, and 1 year **(F)**.

To investigate the development of LCs in duck testes, hematoxylin and eosin (H&E) stains were applied for the morphological observation of the testicular structure among ducks aged 2 months, 6 months, and 1 year. In the 2-month-old duck testes, a great quantity of LCs existed in the testicular interstitium (**Figures 1A, D**). By contrast, modest and inconsiderable amounts of LCs were respectively observed in 6-month-old and 1-year-old duck testes (**Figures 1B, C, E, F**). Additionally, the immunohistochemical analysis indicated that 3β-HSD was highly expressed in the testicular interstitium of 2-month-old ducks (**Figures 2A, D**); however, in contrast, a low expression in 6-month-old and 1-year-old duck testes was observed (**Figures 2B, C, E, F**). Therefore, LCs were noticeable in the testicular interstitium of 2-month-old ducks.

### LC isolation and immunohistochemical analysis from duck testicular interstitium

**Figure 3:**
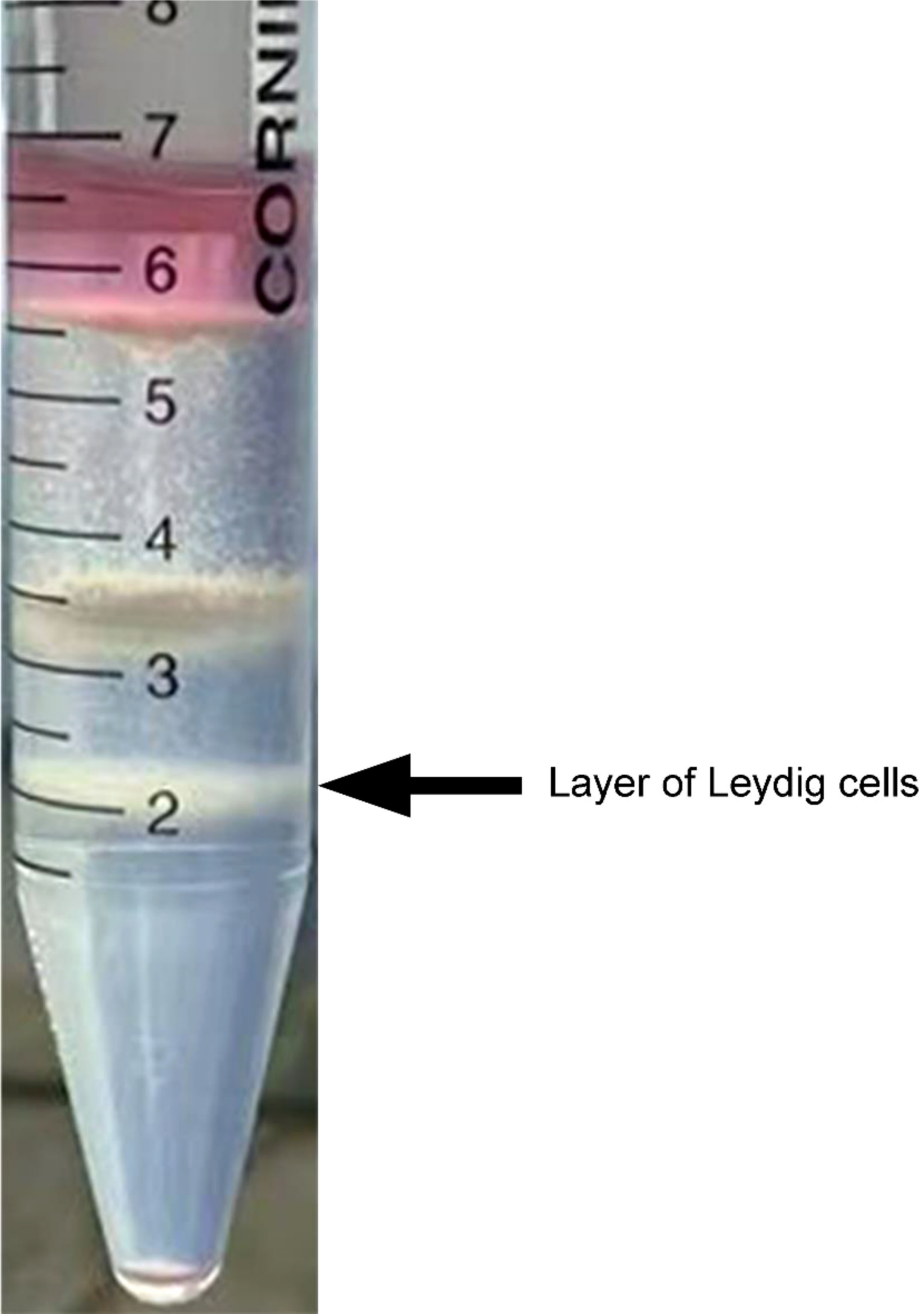
Isolation of Leydig cells applying Percoll density gradient centrifugation. Three cell layers were explicitly separated in the centrifuge tube, and Leydig cells were deposited on the third layer from top to bottom (indicated by the black arrow). In the first or top cell layer, with density of 1.035 g/ml, round cells and cellular debris accounted for the most cell layer. Sperm and interstitial debris constituted the second or middle cell layer, with density of 1.076 g/ml. The third or bottom cell layer, with density of 1.085 g/ml, is composed of Leydig cells.

To acquire highly purified primary duck LCs, collagenase II digestion and Percoll density gradient centrifugation were successively applied to the isolation process. Correspondingly, three cell layers were explicitly separated in the centrifuge tube from top to bottom (**Figure 3**). In the first or top cell layer with a density of 1.035 g/ml, the round cells and cellular debris accounted for the most cell layer. Sperm and interstitial debris constituted the second or middle cell layer with a density of 1.076 g/ml and the third or bottom cell layer with a density of 1.085 g/ml of Leydig cells.

**Figure 4:**
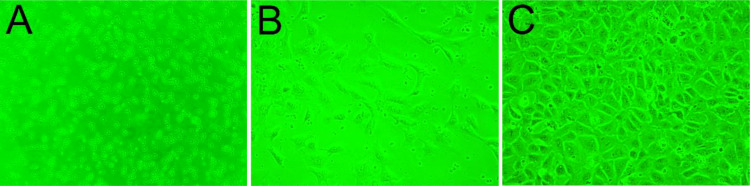
The growth process of primary cultured LCs in different periods. **(A)** Upon inoculation, LCs suspended in serum medium. **(B)** Twelve hours later, LCs in the cell culture flask. **(C)** Nearly 24 h after inoculation, LCs in the cell culture flask. LCs, Leydig cells.

**Figure 5:**
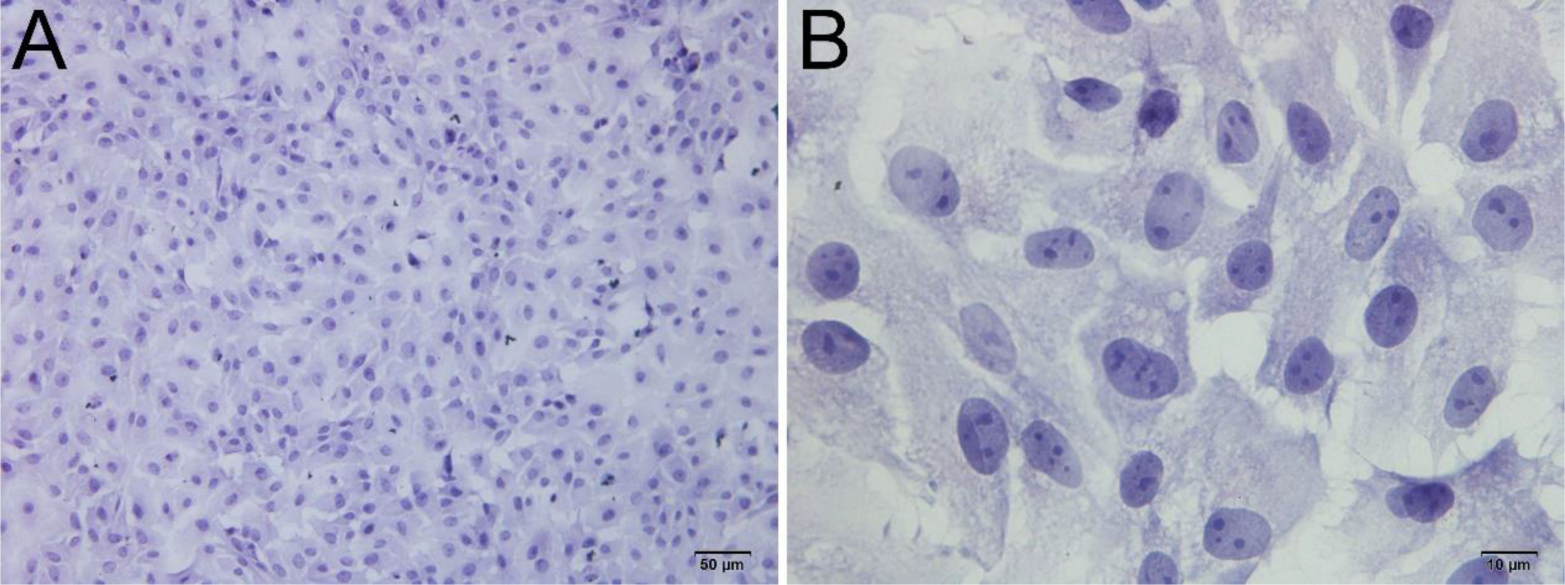
H&E staining of primary cultured LCs at 12 and 24 h. Low magnification **(A)** and high magnification **(B)** of adherent cells. Bar, 50 μm **(A)** and 10 μm **(B)**. LCs, Leydig cells.

**Figure 6:**
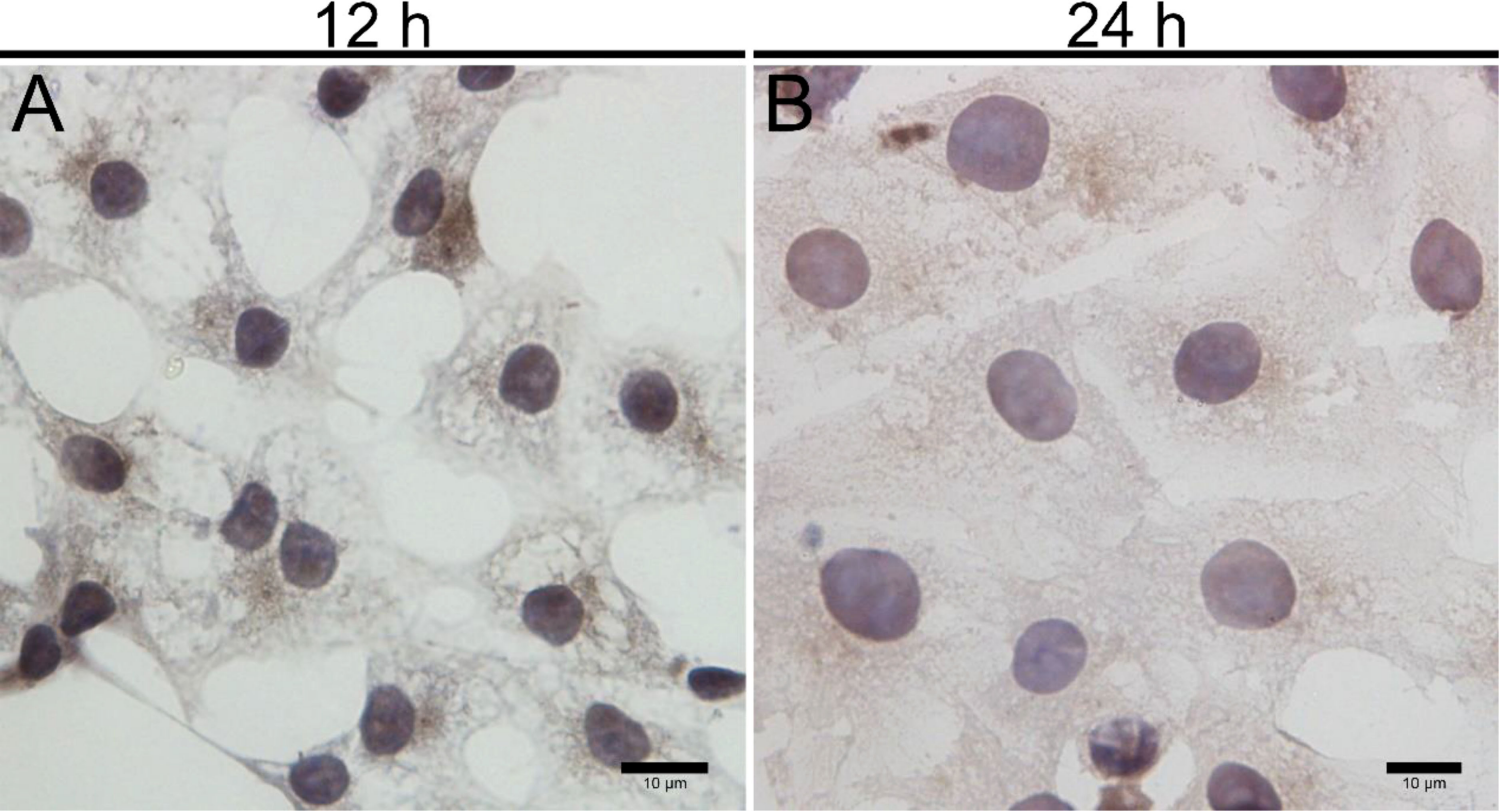
Immunohistochemical identification of 3β-HSD in primary cultured LCs at 12 and 24 h. Low magnification **(A)** and high magnification **(B)** of adherent LCs. Bar, 10 μm **(A, B)**. LCs, Leydig cells.

**Figure 7:**
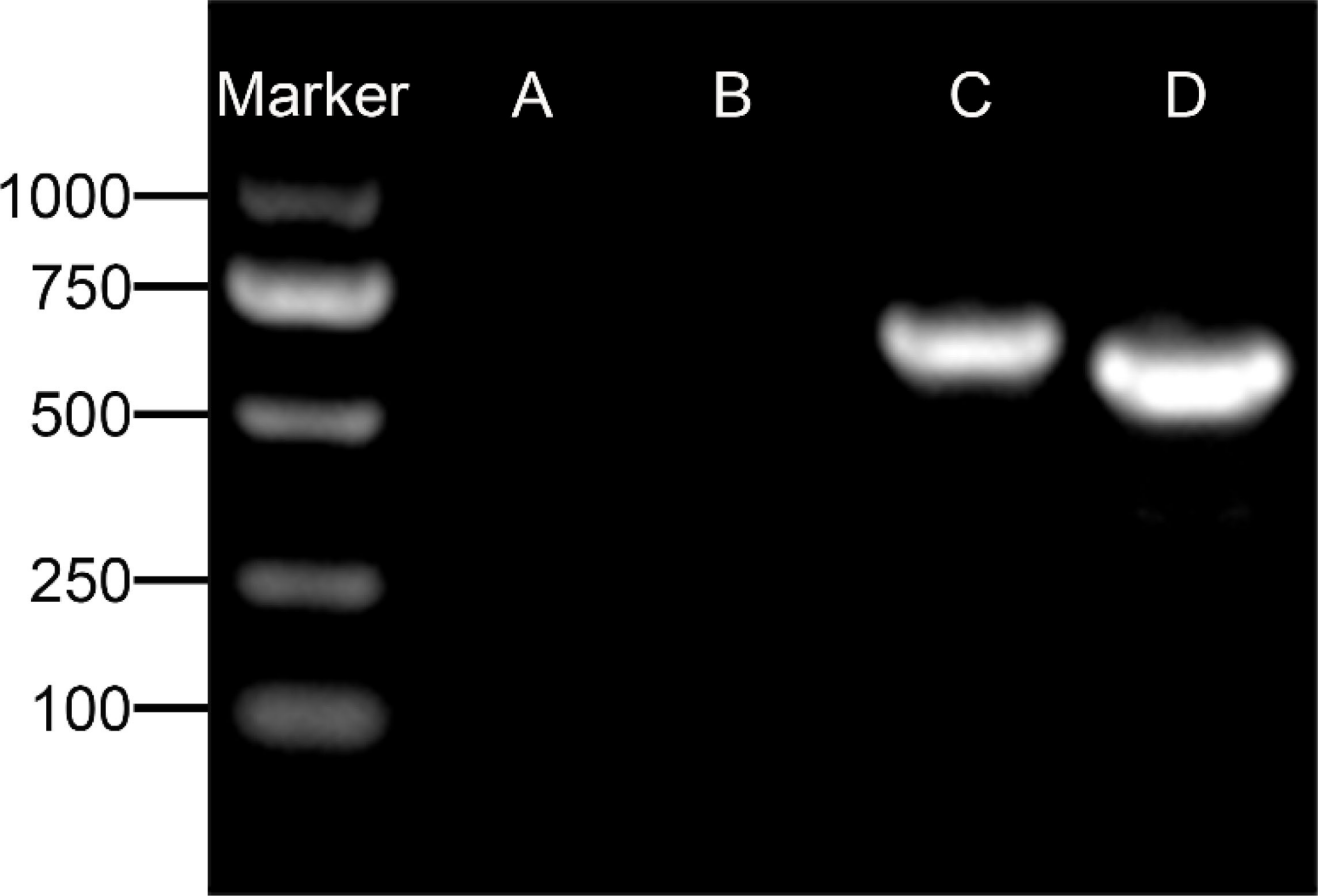
Agarose gel electrophoresis detection of specific gene for spermatogenic cells. **(A)** GCNA-1 for germ cells, **(B)** WT1 for Sertoli cells, **(C)** CYP11A1 for Leydig cells, and **(D)** CYP17A1 for Leydig cells. GCNA-1, germ cell nuclear antigen 1.

Upon inoculation, LCs were round in morphology and suspended in a serum medium (**Figure 4A**). Most of the isolated cells were already adherent and occupied approximately 50% of the cell culture flask after 12 h (**Figure 4B**). Nearly 24 h after inoculation, LCs were adherent in the morphology of cobblestone and cover 80%–90% of the cell culture flask (**Figure 4C**). Additionally, H&E staining indicated that adherent cells were uniform in morphology (**Figure 5A**). The round or oval nuclei were in the center of LCs; meanwhile, small vacuoles (probably lipid droplets) could be observed in the cytoplasm of LCs (**Figure 5B**). To further verify the purity of LCs, the immunohistochemical analysis indicated that 3β-HSD was highly expressed in the cytoplasm of LCs at different stages (**Figure 6**). The specific gene expressions for spermatogenic cells (germ cell nuclear antigen 1 (GCNA-1)), Sertoli cells (WT1 transcription factor (WT1)), and Leydig cells (cytochrome P450 family 11 subfamily A member 1 (CYP11A1) and cytochrome P450 family 17 subfamily A member 1 (CYP17A1)) were observed. In cultured LCs, the expression of GCNA-1 and WT1 were low expressed or undetected, whereas CYP11A1 and CYP17A1 were highly expressed (**Figure 7**).

### Testosterone synthesis and secretion ability in cultured LCs from 2-month-old ducks

**Figure 8:**
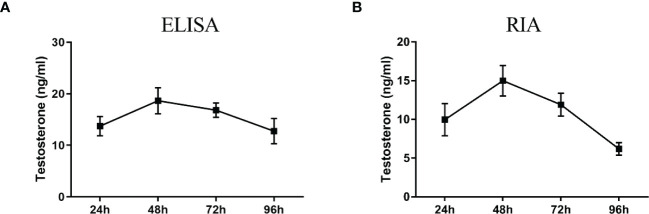
Testosterone detection in primary duck LC supernatant at different periods. ELISA **(A)** and radioimmunoassay (RIA) **(B)** analysis of testosterone level. LC, Leydig cell.

**Figure 9:**
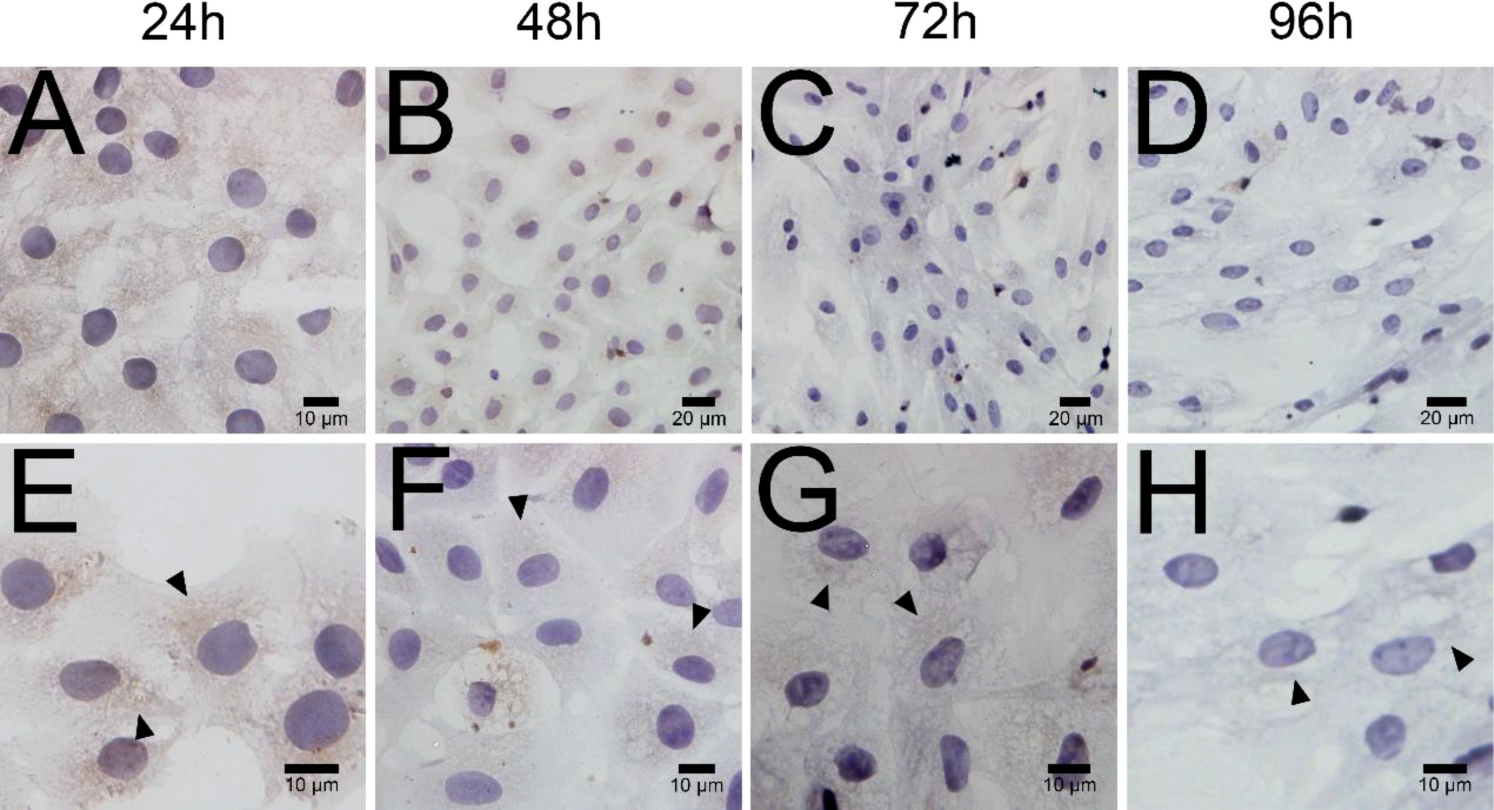
Immunohistochemical analysis of 3β-HSD in primary duck LCs at different periods. Low magnification **(A–D)** of primary duck LCs at 24, 48, 72, and 96 h post culture. High magnification **(E–H)** of primary duck LCs at 24, 48, 72, and 96 h post culture. LCs, Leydig cells.

**Figure 10:**
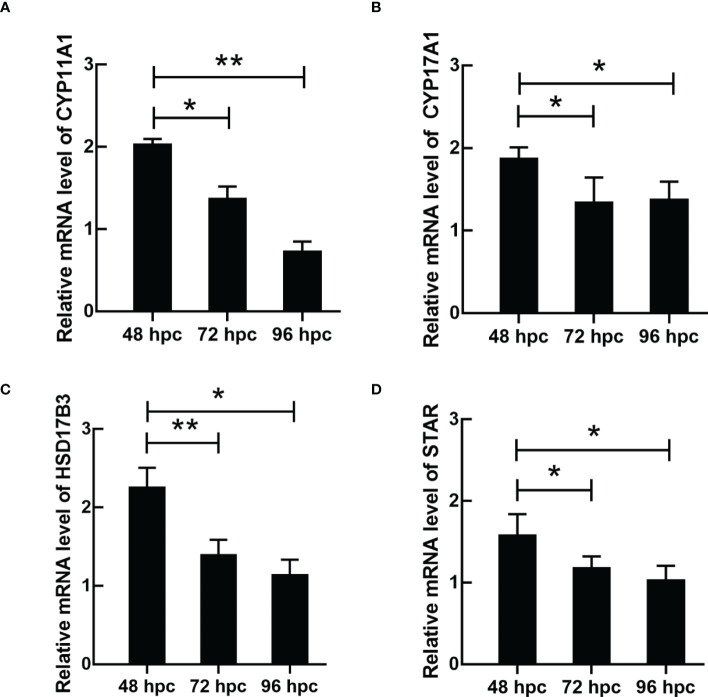
The mRNA expression of testosterone synthesis-related genes in primary duck LCs at different periods. The relative mRNA expression of **(A)** CYP11A1, **(B)** CYP17A1, **(C)** HSD17B3, and **(D)** STAR in LCs at 48, 72, and 96 hac. LCs, Leydig cells. *P<0.01, **P<0.05.

ELISA results revealed that the level of testosterone secreted by primary duck LCs was gradually elevated within the first 48 h and reached the peak 48 h after culture (hac). Subsequently, the testosterone concentration continuously diminished at 72 and 96 hac (**Figure 8A**). Additionally, the testosterone level verified by radioimmunoassay (RIA) is consistent with the ELISA results (**Figure 8B**). Meanwhile, the immunohistochemical analysis indicated that 3β-HSD was highly expressed in 24 hac (**Figures 9A, E**) and 48 hac (**Figures 9B, F**) and decreased gradually in 72 hac (**Figures 9C, G**) and 96 hac (**Figures 9D, H**). The mRNA levels of testosterone synthesis-related genes (CYP11A1, CYP17A1, HSD17B3, and STAR) congruously reached a peak at 48 hac and then reduced at 72 and 96 hac when compared to the mRNA load at 24 hac (**Figure 10**). In consequence, the primary LCs from the ducks (*A. platyrhynchos*) have the typical function—testosterone synthesis and secretion.

## Discussion

In the testis, the seminiferous tubule includes spermatogenic cells and Sertoli cells, which function in spermatogenesis and nourishing spermatogenic cells, respectively. With regard to the LCs in the testicular interstitium, they could synthesize and secrete a male hormone (testosterone) and hence regulate male reproductive function. In animal genetics and breeding, testosterone is of great significance in promoting sexual organ development, spermatogenesis, early stage of follicular development, maintaining secondary sex characteristics, and so on (35). Owing to African swine fever (36), the surging demand for ducks, which were used as a substitute for pork, accelerated the production of ducks. Consequently, the genetics and breeding of ducks, especially the important role of LCs in synthesizing and secreting testosterone, matter for the healthy development of the breeding industry (37). Nevertheless, the isolation, identification, and testosterone synthesis in duck LCs have received little attention. In the present study, highly purified primary LCs were successfully isolated from duck (*A. platyrhynchos*) testes and could stably grow and passage for future research.

In the previous studies, trypsin or collagenase digestion and subsequent Percoll gradient centrifugation were mainly applied to the isolation of animal LCs (28). The testicular tissue was first scattered and digested using trypsin or collagenase. Interestingly, the digestive enzyme applied in different animals was distinct. Collagenase II was used in mice and rats, whereas trypsin and collagenase II mixture was applied to cows (38) and pigs (39). With regard to poultry (such as chicken) (40), successive digestion of trypsin and collagenase II was carried out in the acquisition of cell suspension. In poultry, collagenase II digestion combined with differential centrifugation was applied in the isolation of rooster LCs, and passage was used in its purification (34). The contradistinction in digestive enzyme selection is probably owing to the distinct concentration of connective tissues in viviparous and oviparous animals (41), in which potent trypsin could first digest the connective tissues in chicken testes and then released conglobate seminiferous tubule. In the present study, we undoubtedly followed the previous digestive enzyme selection procedure, accounting for the species similarity between chickens and ducks. Surprisingly, the isolated LCs from duck (*A. platyrhynchos*) testes could not stably grow and passage, in spite of plentiful LC numbers.

To resolve this obstacle, simple trypsin was applied in digestion considering its potent degradative ability on the intercellular junction. Despite the increased cell viability of isolated LCs, the relatively few cell numbers limit its rapid growth and passage. It is quite possible that the robust digestive ability results in weak LC viability, owing to the different reaction responses of distinct tissues/cells on trypsin (42). Finally, gentle collagenase II for longstanding digestion was carried out in the acquisition of cell suspension. Accompanied by mechanical blowing, energetic LCs were isolated and could stably grow. Alternatively, the limited quantity of LCs restricted the passage and future functional analysis of LCs. This extraordinary result raised our doubt that the mature duck testes contained a relatively small number of LCs (43). Given that, the testicular development of various day-age ducks was successively considered. Previously, many studies have focused on Leydig cell development in fetal and adult testes, and the hyperdynamic fetal Leydig cell (FLC) occurs throughout the *in utero* life, peaks during birth, gradually declines, and subsequently disappears during neonatal/pre-pubertal life (18). In the present study, first, we compared the testicular structure among ducks aged 2 months, 6 months, and 1 year via H&E staining, finding that LCs were noticeable in the testicular interstitium of 2-month-old ducks. The 1-year-old duck test used in the previous LC culture identified relatively few and constant numbers of LCs. These above results indicated that LCs in 2-month-old ducks are hyperdynamic and of content. Alternatively, the distinctive LC viability in the testes of 2-month-old ducks could be down to precursor cell differentiation and LC mitosis occurring concurrently (44). Consequently, the testes of 2-month-old ducks underwent LC culture accompanied by prolonged digestion for 1–1.5 h.

In the present study, the function of LCs (especially testosterone synthesis) in ducks (*A. platyrhynchos*) was further investigated. Testosterone synthesis in LCs was regulated by the hypothalamic–pituitary–gonadal axis (HPGA) and materialized by cholesterol (1). After cholesterol translocation from the mitochondrial outer membrane to the mitochondrial inner membrane (45), cholesterol was transformed into pregnenolone via CYP11A1 and subsequently transferred to the endoplasmic reticulum (46), following the pregnenolone–progesterone–androstenedione–testosterone transformation via enzymolysis and catalysis step by step (47). Therefore, testosterone synthesis is a precisely regulated process (48), and insufficient or excessive levels of testosterone could affect body health. Testosterone deficiency would cause male infertility (49), whereas high levels of testosterone could lead to male precocious puberty, adrenal disease, testicular disease, and so on (50). In the present study, the mRNA expression of testosterone synthesis-related genes (CYP11A1, CYP17A1, HSD17B3, and STAR) congruously reached a peak at 48 hac and gradually reduced at 72 and 96 hac, which indicated a periodic regulation of testosterone synthesis. Thus, the studies related to LC function would be more appropriate in LCs cultured after 48 h.

To summarize, highly purified primary LCs were isolated and identified from 2-month-old duck (*A. platyrhynchos*) testes. Meanwhile, the testosterone synthesis ability and related genes were investigated in primary cultured duck LCs. These findings brought us closer to understanding duck LC culture and its application in animal reproduction.

## Conclusion

This study established a feasible method for isolating highly purified primary duck LCs and identified its biological function in synthesizing testosterone. These findings brought us closer to understanding duck LC culture and its application in animal reproduction. Meanwhile, the study laid the foundation for the genetics, breeding, and development of the breeding industry of ducks.

## Data availability statement

The original contributions presented in the study are included in the article/**Supplementary Material**. Further inquiries can be directed to the corresponding authors.

## Ethics statement

The animal study was reviewed and approved by The Science and Technology Agency of Jiangsu Province and Nanjing Agricultural University Veterinary College.

## Author contributions

The authors have made the following declarations about their contributions: XC and XG conceived and designed the experiments. SJ designed this research. XC performed the experiments. AJ and MA analyzed the data. XC and XG wrote the paper. All authors contributed to the article and approved the submitted version.
