# Supplementary material for: Primary culture and endocrine functional analysis of Leydig cells in ducks (Anas platyrhynchos)
Source: Front Endocrinol (Lausanne). 2023 Jun 6;14:1195618. doi: 10.3389/fendo.2023.1195618 (PMC10280297; doi:10.3389/fendo.2023.1195618)
Supplement: Supplementary file 1 [file Table_1.docx]

**Supplement Table 1** Primers for RT-qPCR analysis in *Anas platyrhynchos*.

| **Gene name** | **Sense primer (5'-3')** | **Antisense primer (5'-3')** |
| --- | --- | --- |
| *GCNA* | ccaagcccaacagaaaacat | ggcagttctgggtgaatcat |
| *WT* | cccagcttgaatgcatgac | tgcagtttagtcatgttcctctg |
| *CYP11A1* | ctgaccctgaacaaggaggt | gggtgctcttcagcatcttc |
| *CYP17A1* | caccgaacacaaggaaacct | gaggaacagctccatcttgg |
| *HSD17B3* | cagggcaaagggaattatca | aggcggtcactgtggaatac |
| *STAR* | caacggagacaaagtgctga | caccgtgtctttcccaatct |
